# European Society of Coloproctology guideline on training in robotic colorectal surgery (2024)

**DOI:** 10.1111/codi.16904

**Published:** 2024-03-01

**Authors:** Samson Tou, Stephanie Au, Cillian Clancy, Steven Clarke, Danielle Collins, Frances Dixon, Elizabeth Dreher, Christina Fleming, Anthony G. Gallagher, Marcos Gomez‐Ruiz, Jos Kleijnen, Yasuko Maeda, Katie Rollins, Klaus E. Matzel

**Affiliations:** ^1^ Department of Colorectal Surgery University Hospitals of Derby and Burton NHS Foundation Trust Derby UK; ^2^ School of Medicine University of Nottingham Derby UK; ^3^ NHS Education for Scotland Edinburgh UK; ^4^ Department of Colorectal Surgery Tallaght University Hospital Dublin Ireland; ^5^ University Hospitals of Derby and Burton NHS Foundation Trust Derby UK; ^6^ Department of Colorectal Surgery Western General Hospital, NHS Lothian Edinburgh Scotland; ^7^ Department of Colorectal Surgery Milton Keynes University Hospital NHS Foundation Trust Milton Keynes UK; ^8^ Department of Urology University Hospitals of Derby and Burton NHS Foundation Trust Derby UK; ^9^ Department of Colorectal Surgery University Hospital Limerick Limerick Ireland; ^10^ ORSI Academy Melle Belgium; ^11^ Colorectal Surgery Unit, General Surgery Department Marqués de Valdecilla University Hospital Santander Spain; ^12^ Valdecilla Biomedical Research Institute (IDIVAL) Santander Spain; ^13^ Kleijnen Systematic Reviews Ltd York UK; ^14^ School for Public Health and Primary Care (CAPHRI) Maastricht University Maastricht the Netherlands; ^15^ Department of Surgery Queen Elizabeth University Hospital Glasgow UK; ^16^ Gastrointestinal Surgery, Nottingham Digestive Diseases Centre National Institute for Health Research (NIHR) Nottingham Biomedical Research Centre Nottingham UK; ^17^ Section of Coloproctology, Department of Surgery University of Erlangen‐Nürnberg, FAU Erlangen Germany

**Keywords:** colorectal surgery, competency, curriculum, proficiency, robotics, robotic surgery, training

**Table:** 

EXECUTIVE SUMMARY	777
Recommendations	777
Knowledge required for performing robotic colorectal surgery: recommendations	777
Technical skills required for performing robotic colorectal surgery: recommendations	778
Nontechnical skills required for performing robotic colorectal surgery: recommendation	778
Assessment of competency/proficiency during training in robotic colorectal surgery: recommendation	778
Credentialing in robotic colorectal surgery: recommendation	778
Clinical outcome data registry in robotic colorectal surgery: recommendation	778
BACKGROUND	778
Target audience	778
METHOD	779
Formation of the guideline working group	779
Process of guideline construction	779
Scope of this guideline	779
Knowledge required for performing robotic colorectal surgery	779
Technical skills required for performing robotic colorectal surgery	780
Nontechnical skills required for performing robotic colorectal surgery	780
Assessment of competency/proficiency during training in robotic colorectal surgery	780
Credentialing in robotic colorectal surgery	780
Clinical outcome data registry in robotic colorectal surgery	780
Outcomes measured	780
Critical for decision‐making	781
Important, but not critical for decision‐making	781
Of low importance	781
Literature searches	781
Resources	781
Handling of citations	781
Study eligibility assessment and selection	781
Reviewing research evidence	781
Method used to make recommendations	782
Drafting of statements and supporting text	782
RESULTS	782
Knowledge required for performing robotic colorectal surgery	782
Recommendation	782
Rationale for recommendation	782
Future research priorities	782
Recommendation	782
Rationale for recommendation	783
Future research priorities	783
Technical skills required for performing robotic colorectal surgery	783
Recommendation	784
Rationale for recommendation	784
Learning curve measured in terms of clinical outcomes	784
Conversion	784
Postoperative complications	784
GRADE quality assessment on conversion and postoperative complications	785
Other clinical outcomes	786
Operating time	786
Anastomosis‐related complications	786
Learning curve measured in terms of simulator performance	786
Future research priorities	786
Recommendation	786
Recommendation	786
Rationale for recommendation	787
Operative performance	787
Patient outcome	787
Future research priorities	788
Recommendations	788
Rationale for recommendation	788
Future research priorities	788
Recommendations	788
Rationale for recommendation	788
Future research priorities	789
Recommendations	789
Rationale for recommendation	789
Safety of adopting modular training	789
Future research priorities	789
Recommendations	790
Rationale for recommendation	790
Future research priorities	790
Recommendations	790
Rationale for recommendation	790
Future research priorities	790
Recommendations	791
Rationale for recommendation	791
Future research priorities	791
Recommendations	791
Rationale for recommendation	792
Future research priorities	792
Recommendations	792
Rationale for recommendation	792
Future research priorities	792
DISCUSSION	792
Research gaps	794
CONCLUSION	794
REVIEW AND QUALITY ASSURANCE	794
AUTHOR CONTRIBUTIONS	794
ACKNOWLEDGEMENTS	795
FUNDING INFORMATION	795
CONFLICT OF INTEREST STATEMENT	795
DATA AVAILABILITY STATEMENT	795
ETHICAL APPROVAL AND INFORMED CONSENT	795
DEFINITIONS AND GLOSSARY	795
REFERENCES	796
APPENDIX	799

## EXECUTIVE SUMMARY

Robotic surgery has been utilized increasingly, including in colorectal surgery. Newer robotic platforms are coming onto the market, and more emphasis is being placed on the safety and adequate training of surgeons and theatre teams. Training in robotic colorectal surgery has not been standardized, and there are no agreed structured training and assessment methods. Some studies in minimally invasive surgery across specialities have shown that training curricula shortened the learning curve in minimally invasive surgery and, therefore, there is a greater need for guidance on training in robotic colorectal surgery based on up‐to‐date available evidence on the subject.

The European Society of Coloproctology (ESCP) Guidelines Committee aimed to conduct a comprehensive literature review, assess currently available evidence and collate expert opinion on training in robotic colorectal surgery. Evidence was graded, and the recommendation was based on the GRADE (Grading of Recommendations Assessment, Development and Evaluation) methodology. When evidence is lacking expert opinion is considered, and the research gap is highlighted.

### Recommendations

The robotic guideline group addressed six topics with 15 research questions in the PICO format (patient/population, intervention, comparison and outcomes) and developed 11 recommendations.

Most of the recommendations are based on a low or very low quality of evidence. Where the case benefits could be seen by indirect evidence or strong recommendations are unwarranted but made as good practice statements, they are made explicit by stating ‘expert opinion only’ without a GRADE level.

#### Knowledge required for performing robotic colorectal surgery: recommendations

- Robotic platform training is essential to patient safety and therefore should be used in a structured colorectal robotic training curriculum. [Expert opinion only]
- eLearning could be used to deliver content in colorectal robotic surgery training. [Expert opinion only]

#### Technical skills required for performing robotic colorectal surgery: recommendations

- Prior laparoscopic experience is not essential for training in robotic colorectal surgery. [Very low quality of evidence; *conditional recommendation*]
- No recommendation could be provided regarding the effects of having prior experience of one robotic platform versus no such prior experience on the learning curve for learning another robotic platform.
- Simulation should be used as part of the colorectal robotic training curriculum. [Very low level of evidence and expert opinion, upgraded by the guideline working group; *strong recommendation*]
- In‐person intraoperative mentoring should be used for surgeons performing their initial colorectal robotic cases. [Expert opinion only]
- Telementoring could be considered to support mentoring in training colorectal robotic surgery in selected cases. [Very low level of evidence; *conditional recommendation*]
- A modular approach to procedural training in the operating room could be considered in robotic colorectal surgery training. [Very low level of evidence and expert opinion; *conditional recommendation*]
- A structured train‐the‐trainer (TTT) course could be considered for robotic colorectal surgery. [Very low level of evidence and based on expert opinion; *conditional recommendation*]

#### Nontechnical skills required for performing robotic colorectal surgery: recommendation

- Nontechnical skills training could be considered as part of a colorectal training curriculum. [Very low level of evidence; *conditional recommendation*]

#### Assessment of competency/proficiency during training in robotic colorectal surgery: recommendation

- No recommendation could be provided on the effects of competency‐, proficiency‐based supervised training (for learners) versus noncompetency‐, nonproficiency‐based supervised training during colorectal robotic surgery training on operative performance and patient clinical outcomes.

#### Credentialing in robotic colorectal surgery: recommendation

- Credentialing could be considered for defining requirements in commencement and maintenance of robotic colorectal surgical practice. [Very low level of evidence and based on expert opinion; *conditional recommendation*]

#### Clinical outcome data registry in robotic colorectal surgery: recommendation

- Registering clinical outcome data could be considered in a structured colorectal robotic training curriculum. [Expert opinion only]

## BACKGROUND

The use of robotic surgery has steadily increased over the last years in both general surgery and colorectal surgery [1]. Approximately 1000 robotic‐assisted procedures were performed worldwide in 2000; by 2018 this had increased to more than a million [2]. The advantages of robotic surgery were thought to be its suitability for confined spaces and complex operations such as rectal cancer surgery. The application and volume of practice continue to expand, and more robotic platforms are coming to the market [1, 3]. The projected global surgical robot market by 2025 is 275 billion USD. This is driven by innovation, growth in procedure volume and access to emerging markets [4].

It is crucial when introducing surgical techniques that patients should not come to harm, and surgical societies should have a leading role in appraising evidence and implementing surgical procedures [5, 6]. Evidence has suggested that training curricula shortened the learning curve in laparoscopic surgery and robotic surgery [7, 8]. However, there are variations in training components and assessments in different curricula [9]. It is therefore crucial to appraise the evidence on some key training components when implementing a structured training programme.

This guideline sets out to:

- review the evidence for components of robotic colorectal training using GRADE methodology;
- review the evidence concerning the different assessments used and quality control in robotic colorectal training using GRADE methodology;
- identify the gaps in evidence and research in robotic colorectal training;
- examine the implications for future robotic colorectal training.

### Target audience

This guideline is written and intended for surgeons, theatre teams, trainees, purchasers, local, regional and national policymakers, hospital leaderships, scientific societies, professional bodies for training and accreditation, and industry partners.

## METHOD

### Formation of the guideline working group

The ESCP guidelines committee appointed project leads (ST, YM, DC) to curate this guideline. A steering group was formed with experts in robotic surgery, training and education, and guideline development with a common interest in improving training in robotic colorectal surgery. ESCP e‐newsletters and social media announced a call for other working group members to participate in the guideline. The selection of final working group members was assessed based on the following set of criteria, and also keeping to the principle of equality, diversity and inclusion (EDI): 1. Appropriate and relevant clinical experience.2. A proven track record of scientific knowledge and research skills.3. International expertise and recognition or willingness to collaborate with diverse professionals and patients.4. Geographical distribution.

The working group comprises colorectal surgeons, trainees, educators, expert robotic surgeons, surgical assist/allied health professionals familiar with robotic training, a patient representative and GRADE methodologist. A professor in systematic reviews and expert guideline methodologist helped with the methodological aspects of this guideline (Table 1).

**TABLE 1:** Guideline working group.

Guideline member	Function	Country
Stephanie Au	Surgical registrar with special interest in guideline development	United Kingdom
Steven Clarke	Patient representative	United Kingdom
Cillian Clancy	Consultant colorectal surgeon with special interest in systematic review	Ireland
Danielle Collins	Consultant colorectal surgeon with special interest in robotic surgery	United Kingdom
Frances Dixon	Surgical registrar with special interest in robotic surgery	United Kingdom
Elizabeth Dreher	Specialist nurse and first assist, robotic surgery with special interest in training theatre personnel	United Kingdom
Christina Fleming	Consultant colorectal surgeon with special interest in robotic surgery	Ireland
Anthony Gallagher	Director of research and skill development with special interest in proficiency‐based training	Northern Ireland
Marcos Gómez Ruiz	Consultant colorectal surgeon with special interest in robotic surgery	Spain
Jos Kleijnen	Professor of systematic reviews and expert guideline methodologist	The Netherlands
Yasuko Maeda	Consultant colorectal surgeon with special interest in guideline development methodology	United Kingdom
Klaus Matzel	Consultant colorectal surgeon with special interest in training in robotic colorectal surgery	Germany
Katie Rollins	Surgical registrar with special interest in systematic review	United Kingdom
Samson Tou	Consultant colorectal surgeon with special interest in robotic colorectal surgery	United Kingdom

The group worked closely with the methodologist (JK) to devise a strategy to perform a single set of searches to address all statements and questions relating to training in robotic colorectal surgery. The searches were not limited by date, language or publication status. The group assessed the evidence with robust analysis using GRADE. The current guidance analysed all available data through GRADE so that the strengths and limitations are transparent, and the grade of recommendation is based on these analyses.

### Process of guideline construction

This guideline development followed the ESCP guideline recommendations and the AGREE II tool [10].

### Scope of this guideline

This guideline focuses on the common training components, assessments and quality controls used in robotic colorectal training. Therefore it does not cover robotic surgery in other specialities nor other minimally invasive techniques in colorectal surgery.

This guideline aims to address the PICO (Patient/Population/Problem, Intervention, Comparison, Outcome) questions detailed in the following subsections.

#### Knowledge required for performing robotic colorectal surgery

What are the effects of robotic platform training (for learners) versus no robotic platform training on patient safety in robotic colorectal surgery?

*What are the effects of procedural anatomy training (for learners) versus *no procedural anatomy training on patient safety in colorectal robotic surgery training?*

*What are the effects of the modular approach on procedural training (for learners) versus *not using the modular approach on the learning curve of colorectal robotic surgery training?*

Is eLearning more effective than traditional learning for health professional trainees in colorectal robotic surgery training?

*Note: During the Working Group discussions, these two research questions were initially proposed but dropped: please see the Results section.

#### Technical skills required for performing robotic colorectal surgery

What are the effects of having prior laparoscopic experience (for learners) versus no prior laparoscopic experience on the learning curve for robotic colorectal surgery?

What are the effects of having prior experience of one robotic platform (for learners) versus no such prior experience on the learning curve for learning another robotic platform?

What are the effects of simulation training (for learners) versus no simulation training on operative performance in colorectal robotic surgery?

What are the effects of simulation training (for learners) versus no simulation training on patient outcomes in colorectal robotic surgery?

What are the effects of using mentoring (for learners) versus no mentoring on clinical outcomes in training colorectal robotic surgery?

What are the effects of telementoring versus onsite mentoring (for learners) on clinical outcomes in colorectal robotic surgery?

What are the effects of the modular approach on procedural training in the operating room (for learners) versus not using the modular approach on the learning curve of colorectal robotic surgery training?

What are the effects of attending a structured TTT course for robotic surgery (for trainer) versus not attending such a course on the operative performance (trainee) of colorectal robotic surgery training?

#### Nontechnical skills required for performing robotic colorectal surgery

What are the effects of nontechnical skills training (for learners) versus no nontechnical skills training on patient safety in colorectal robotic surgery?

#### Assessment of competency/proficiency during training in robotic colorectal surgery

What are the effects of competency‐, proficiency‐based supervised training (for learners) versus noncompetency‐, nonproficiency‐based supervised training during colorectal robotic surgery training on operative performance?

What are the effects of competency‐, proficiency‐based supervised training (for learners) versus noncompetency‐, nonproficiency‐based supervised training during colorectal robotic surgery training on patient clinical outcomes?

#### Credentialing in robotic colorectal surgery

What are the effects of credentialing (for the practitioner) versus no credentialing in colorectal robotic surgery on patient clinical outcomes?

#### Clinical outcome data registry in robotic colorectal surgery

What are the effects of registering clinical outcome data (for the practitioner) versus no registering in colorectal robotic surgery on patient clinical outcomes?

### Outcomes measured

A list of outcome measurements was suggested by members of the working group relevant to the PICO questions. Some outcomes are more important in certain PICOs than others. As there are many PICO questions, these outcomes were grouped into the following categories.

Patient safety/patient outcomes/clinical outcomes:

‐ intraoperative
‐ postoperative◦ Clinical◦ Oncological◦ Functional◦ Quality of life

Learning curve:

‐ operative skills
‐ surrogate markers◦ Time◦ Complication rates◦ Oncological outcomes

Effectiveness:

‐ health professionals’ behaviour, skills or knowledge

Operative performance:

‐ time to complete a task
‐ complications
‐ errors
‐ procedural steps completed
‐ validated scores◦ Global Assessment Score (GAS), Global Rating Scale (GRS)◦ Global Evaluative Assessment of Robotic Skills (GEARS)◦ objective performance metrics such as proficiency‐based progression (PBP) metrics

According to the GRADE recommendations [11], outcomes were ranked according to their relative importance into three groups by panel members: (1) critical for decision‐making; (2) important, but not critical for decision‐making; (3) of low importance. The number next to the outcomes is on an importance scale (e.g. 1 is least important and 9 is most important). Outcomes in the first two categories, i.e. (1) and (2), will be included in the evidence profile.

#### Critical for decision‐making

- Mortality (9)
- Intraoperative complications (8)
- Postoperative complications (7)

#### Important, but not critical for decision‐making

- Learning curve (6)
- Conversion (5)
- Operative performance (4)
- Health professionals' behaviour, skill or knowledge (3)

#### Of low importance

- Length of hospital stay (2)
- Readmission (1)

### Literature searches

Literature searches were conducted on 4 May 2022 to identify relevant references on training for robotic colorectal surgery. The search strategy was supported by an expert methodologist (JK) in systematic reviews and guidelines and his team. A single set of searches was devised which aimed to address all statements and questions raised in this topic area. The search strategies were developed specifically for each database and the keywords adapted according to the configuration of each database. Searches were not limited by date, language or publication status.

### Resources

The following databases were searched:

- Embase (Ovid): 1974 to 3 May 2022
- MEDLINE and Epub ahead of print, in‐process, in‐data‐review and other non‐indexed citations and daily (Ovid): 1946 to 3 May 2022
- CINAHL (EBSCO): 1981 to 4 May 2022
- Cochrane Database of Systematic Reviews (CDSR) (Wiley): issue 4 of 12, April 2022
- Cochrane Central Register of Controlled Trials (CENTRAL) (Wiley): issue 4 of 12, April 2022
- KSR Evidence (Internet) (https://ksrevidence.com/): to 4 May 2022

A further up‐to‐date search was performed on 1 September 2023.

Full details of all search strategies are presented in Appendix 0.

### Handling of citations

References identified from the searches were downloaded into EndNote bibliographic management software for further assessment and handling.

### Study eligibility assessment and selection

Two guideline authors (ST, KR) reviewed all the abstracts generated from the searches stored in the database and retrieved the full papers for the potential studies. The two guideline authors independently identified studies, resolving disagreements through discussion with the guideline group. References in the included studies were assessed for any suitable articles for inclusion and any additional studies identified by the working group. For each predefined review question, we included study(ies) with the best available evidence, and these include randomized controlled studies, comparative studies, case series, reviews and expert opinions. Guideline authors were not blind to authors' names, institutions or journals.

### Reviewing research evidence

For each included study, data extraction was based on predefined outcomes. If the data were available, we aimed to compare the differences in effect between the baseline and after treatment in the treatment group and the difference in baseline and after treatment in the control group. We intended to present the results using confidence interval (CI) with the use of Review Manager (RevMan) 5 (Version 5.4, Copenhagen, The Cochrane Collaboration). Individual study quality was assessed using the GRADE score. Additionally, the quality of the evidence for each question was evaluated with the use of the GRADE system, which assigns one of four levels of evidence: very low (⊕∘∘∘), low (⊕ ⊕ ∘∘), moderate (⊕ ⊕ ⊕∘) or high (⊕ ⊕ ⊕⊕). Within the GRADE system, randomized controlled trials (RCTs) were generally rated as high quality but may have been downgraded on the basis of specific design flaws. Observational studies were generally assigned a low quality but may have been upgraded based on the strength of the association demonstrated and the absence of bias. The outcomes of study assessment are presented using the GradePro Guideline Development Tool (https://gdt.gradepro.org/app/).

### Method used to make recommendations

We used standard terminology to make recommendations based on the level of evidence:

- must be used (high level of evidence)
- should be used (moderate level of evidence)
- could be used (low level of evidence)
- can be considered (very low evidence or lack of evidence). In some instances when there was a very low level of evidence or lack of evidence, the guideline group discussed that, when appropriate, the terminology ‘Can be considered’ could be used.

In some instances where there was no evidence or a low level of evidence we upgraded the statement after discussion within the guideline group. When there was no clear evidence in the literature, yet practice or concept was established with consensus among clinicians, recommendations were made as ‘Expert opinion only’ and distinguished as ‘Upgraded recommendation’.

### Drafting of statements and supporting text

All statements and the initial supporting text were presented via a virtual working group meeting. The content and the strength of each statement and recommendation were further reviewed at a dedicated Guideline Session at the ESCP Annual Conference in Dublin in September 2022. All statements were then revised to meet the changes recommended. Following this meeting, a final working group virtual meeting was convened to finalize all statements. All statements and the supporting text were subsequently edited by ST, YM and KM before the paper was sent for final revision and approval by all the authors combined.

## RESULTS

**FIGURE 1:**
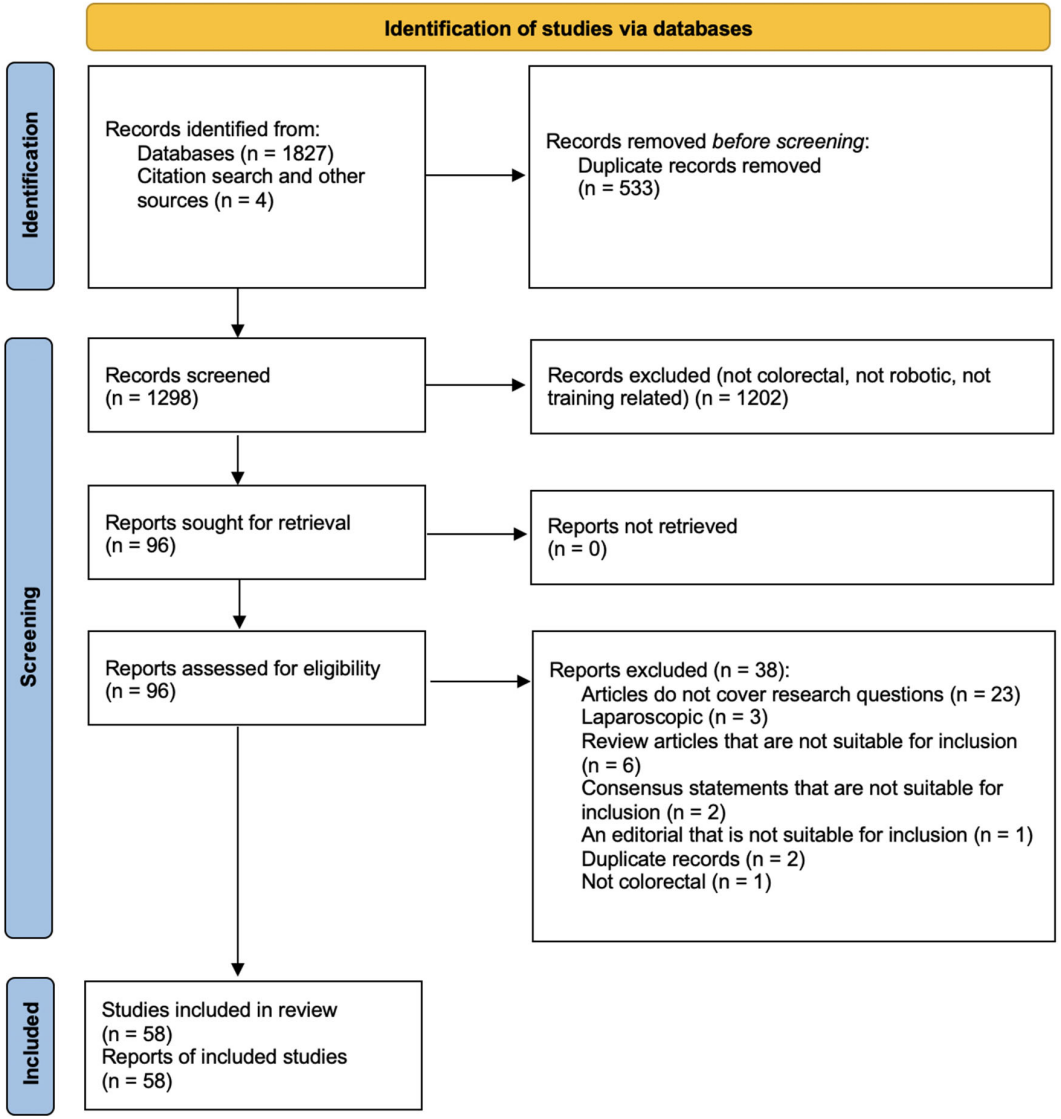
PRISMA flowchart.

The searches retrieved a total of 1831 records. After removing the duplicates, 1298 records remained and were screened by two guideline authors. Fifty‐eight articles were included in this review (Figure 1).

During the discussion among the working group, the question on procedural anatomy training was dropped as this question was best incorporated into eLearning. The modular approach of knowledge learning would be under eLearning/procedural training. However, we may have to reassess these research questions in the future guidance. In the end, the working group generated the following recommendation statements for these research questions.

### Knowledge required for performing robotic colorectal surgery

**Question 1:** What are the effects of robotic platform training (for learners) versus no robotic platform training on patient safety in robotic colorectal surgery?

#### Recommendation

Robotic platform training is essential to patient safety and therefore should be used in a structured colorectal robotic training curriculum. [Expert opinion only]

#### Rationale for recommendation

Very little evidence has addressed this question, as platform training has been accepted as a fundamental element and requirement of robotic surgery training from its inception. Few recent articles have focused on investigating it, including this literature summary. In 2015, Tsuda et al. published a literature review to summarize the clinical evidence of the safety and effectiveness of the da Vinci Surgical System (Intuitive Surgical, Sunnyvale, CA) [12]. The authors summarized peer‐reviewed publications up to 2014 and specifically supported individual platform training.

It is considered best practice that all users undergo individual robotic platform training (basic technology training/basic device training/‘buttonology’) before performing live robotic surgery [13]. Furthermore, it is recommended that training should be completed on each different platform before use. This concept is translated from the aviation industry, where training is required on each aeroplane model before flying and is generally accepted.

#### Future research priorities

There is a paucity of research evidence on the benefits of specific device/console training in robotic surgery. This is gaining increasing importance with the clinical introduction of multiple robotic platforms. The transferability of skills from one platform to another should also be addressed, whether there are benefits of being trained in one platform previously and how it may impact the learning curve. Patient safety, in terms of complications, should be measured according to the type of platform training.

**Question 2:** Is eLearning more effective than traditional learning for health professionals in colorectal robotic surgery training?

#### Recommendation

eLearning could be used to deliver content in colorectal robotic surgery training. [Expert opinion only]

#### Rationale for recommendation

One abstract described a dedicated website to promote surgical learning and training – the Advance in Surgery (AIS) Channel [14]. It was created to provide a learning experience for colorectal and robotic surgery. The teaching is self‐administered without formal feedback. This website is online‐based, delivered by experts and focuses on surgical techniques, anatomy, live surgery and debates.

Two studies [15, 16] examined the educational value of videos of robotic right hemicolectomy posted on YouTube (San Bruno, CA, US). Uzunoglu and colleagues evaluated the educational value of the videos by three experienced oncological surgeons using a Likert scale. They classified these videos into good, moderate or poor according to how many predefined steps these videos contain. Sixty‐eight videos were assessed, and the authors observed that the educational value of these videos was variable. Bal and colleagues conducted a similar study on a YouTube channel assessing robotic right hemicolectomy (with or without complete mesocolic excision). Various methods were used to evaluate the quality and educational value of these videos, including a modified LAP‐VEGaS criterion [17, 18]. Seventy‐two videos were assessed, and most were deemed to have insufficient educational value.

Herrando et al. 2023 [19] published robotic colorectal procedural surgical techniques on the *Colorectal Disease* (the official Journal for the Association of Coloproctology of Great Britain & Ireland and ESCP) YouTube channel. The videos posted via this route have gone through the peer‐reviewed process to enhance their educational value.

#### Future research priorities

There are widely available teaching materials for robotic surgery on the web, some are from individual surgeons and others are from organizations. There is no online quality assurance mechanism for materials, and the industry funds some of these teaching materials. It is crucial to have independent bodies provide objective assessments of the training materials to ensure there is no conflict of interest. These training materials should be developed with clear aims, objectives and assessment components for educational purposes and to assess learners' proficiency in the content. Studies should examine the effectiveness of this eLearning educational content, surgeons or trainees' acceptability and accessibility.

### Technical skills required for performing robotic colorectal surgery

**Question 3:** What are the effects of having prior laparoscopic experience (for learners) versus no prior laparoscopic experience on the learning curve for robotic colorectal surgery?

#### Recommendation

Prior laparoscopic experience is not essential for training in robotic colorectal surgery. [Very low quality of evidence; *conditional recommendation*]

#### Rationale for recommendation

Nine articles were considered relevant to this statement. Three of the nine articles had numerical data comparing the learning curve of trainees with prior laparoscopic colorectal experience with trainees with no such prior experience.

##### Learning curve measured in terms of clinical outcomes

Two studies [20, 21] had data on the learning curve of robotic colorectal surgery measured in terms of clinical outcomes. In this guideline, the clinical outcomes of these two studies have been pooled to facilitate comparison.

Noh et al. (2020) was a retrospective observational study on 662 patients who underwent robotic low anterior resection for low rectal cancer [20]. They were stratified into five groups according to operating surgeons with varying laparoscopic experience (from a previous 403 laparoscopic cases to no cases) and their clinical outcomes were analysed.

Sian et al. (2018) was an observational study on the clinical outcomes of the first 30 robotic colorectal procedures (including high anterior resection, low anterior resection, abdominoperineal resection, right hemicolectomy and abdominal suture rectopexy) performed by two surgeons, one who was a minimally invasive colorectal trained surgeon (T) and the other was a nonminimally invasive colorectal trained surgeon (nT) [21]. There were no statistical comparisons performed between the two comparators.

#### Conversion

**FIGURE 2:**
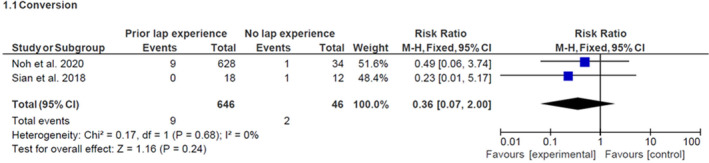
Results for conversion rate (prior laparoscopic experience versus no prior laparoscopic experience).

Both studies have data on conversion rate and the results are summarized in Figure 2. There was no statistically significant difference in conversion between surgeons with prior laparoscopic experience and those without (*p* = 0.24).

#### Postoperative complications

**FIGURE 3:**
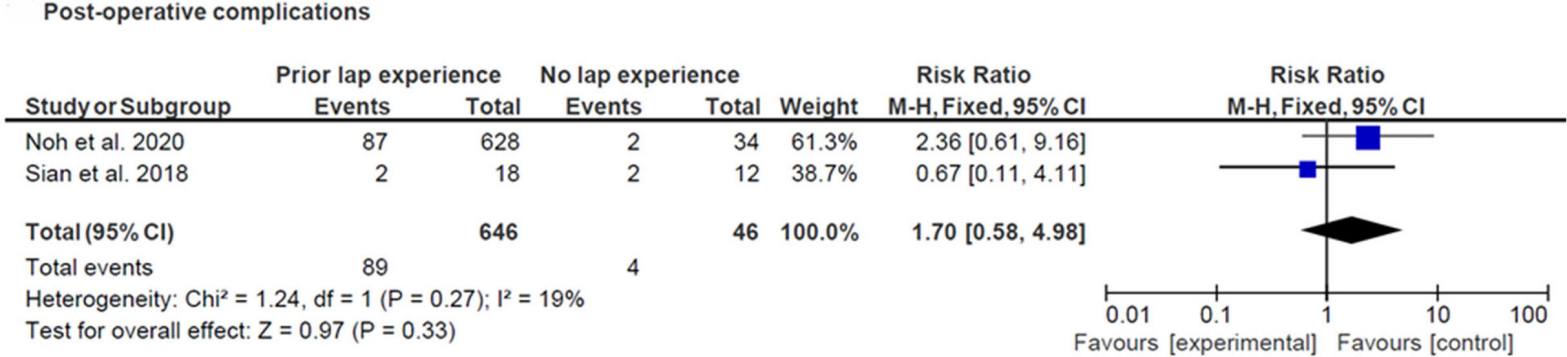
Results for post‐operative complications (prior laparoscopic experience versus no prior laparoscopic experience).

Both studies have data on postoperative complications and the results are summarized in Figure 3. There was no statistically significant difference in postoperative complications between surgeons with prior laparoscopic experience and those without (*p* = 0.33). However, it should be noted that while Sian et al. (2018) [21] had provided a description of postoperative complications included in their study, namely wound infection, pelvic collection, wound dehiscence and postoperative bleeding, Noh et al. (2020) [20] had not provided a breakdown of the postoperative complications observed.

#### GRADE quality assessment on conversion and postoperative complications

GRADE quality assessment for Noh et al. (2020) [20] and Sian et al. (2018) [21] on conversion and postoperative complications are summarized in Table 2. Both outcomes are graded very low in certainty due to risk of selection bias, confounding bias, inconsistency and imprecision.

**TABLE 2:** GRADE assessment. No suitable comparative studies examined the research questions other than Question 3 (prior laparoscopic experience versus no prior laparoscopic experience). Therefore only the GRADE table for Question 3 is included.

Certainty assessment							No. of patients		Effect		Certainty	Importance
No. of studies	Study design	Risk of bias	Inconsistency	Indirectness	Imprecision	Other considerations	Prior laparoscopic experience	No prior laparoscopic experience	Relative (95% CI)	Absolute (95% CI)		
*Conversion*												
2	Observational studies	Serious^a^ ^,^ ^b^ ^,^ ^c^ ^,^ ^d^	Serious^e^	Not serious	Serious^f^	None	9/646 (1.4%)	2/46 (4.3%)	**RR 0.36** (0.07 to 2.00)	**28 fewer per 1000** (from 40 fewer to 43 more)	⨁◯◯◯ Very low	Important
*Post‐operative complications*												
2	Observational studies	Serious^a^ ^,^ ^b^ ^,^ ^c^ ^,^ ^d^	Serious^g^	Not serious	Serious^h^	None	89/646 (13.8%)	4/46 (8.7%)	**RR 1.70** (0.58 to 4.98)	**61 more per 1000** (from 37 fewer to 346 more)	⨁◯◯◯ Very low	Critical

Abbreviations: CI: confidence interval; RR: risk ratio.

^a^ In both studies there was no randomization of patients, no blinding and no allocation concealment.

^b^ In Noh et al. each surgeon had their own selection criteria for patients undergoing robotic surgery, thereby introducing selection bias.

^c^ In Sian et al. patients were allocated based on list availability. The study has a low number of patients in total (30) and the minimally invasively trained surgeon (T) seemed to have more patients (18) than the nonminimally invasive trained surgeon (nT) (12). There seemed to be an unequal case mix for the two surgeons. nT seemed to have more low anterior resection cases and had also performed right‐hemicolectomies. The author also admitted that patients were carefully selected as the institution had just initiated robotic surgery. These points raise the possibility of selection bias.

^d^ In Noh et al. the five surgeons did not start robotic surgery at the same time, with the surgeons with more laparoscopic experience having started first. Therefore subsequently trained surgeons, who had less or no laparoscopic experience, might have benefited from observing and monitoring the initials experience of the previously trained robotic surgeons. Also, late adopters could have been offered more training programmes, such as dry lab, than early adopters. These points raise the possibility of confounding bias.

^e^ The CIs of the two studies overlap and *I* ^2^ is 0% (*p* = 0.68). However, the study designs are quite heterogeneous, with one study looking at low anterior resection only while the other looks at a range of colorectal procedures.

^f^ The CIs of both studies are quite considerable (0.06–3.74 and 0.01–5.17). Also, one study is rather small with a sample size of 30.

^g^ The CIs of the two studies overlap and *I* ^2^ is 19% (*p* = 0.27). However, the study designs are quite heterogeneous, with one study looking at low anterior resection only while the other looks at a range of colorectal procedures.

^h^ The CIs of both studies are quite substantial (0.61–9.16 and 0.11–4.11). Also, one study is rather small with a sample size of 30.

#### Other clinical outcomes

##### Operating time

Noh et al. (2020) [20] provided the mean operating time while Sian et al. (2018) [21] provided the median operating time, therefore their results cannot be pooled together. In Noh et al, the average operating time for surgeons with previous laparoscopic experience was 303.09 min and that of the surgeon with no prior laparoscopic experience was 305.1 min. In Sian et al., the median operating times of T and nT were respectively 5 h and 5.5 h for high anterior resection, 7 and 5.5 h for low anterior resection and 8 and 4 h for abdominoperineal resection.

The learning curve in terms of operating time was analysed in Noh et al. [20] using the cumulative sum technique. Surgeon A with the greatest experience of laparoscopic rectal surgery showed a learning curve period of 110 cases. Surgeons B and C, who had less laparoscopic experience, had learning curves of 39 and 114 cases, respectively, while surgeons D and E, with limited laparoscopic surgery experience, had learning curves of 55 and 23 cases, respectively. One potential confounding factor could be the different timing in initiating robotic surgery, with Surgeons A and C being early adopters. Surgeons B, D and E were later adopters and had a shorter learning curve. This might be because they had benefited from observing A's and C's experience and might have been offered more tips and training programmes before initiation.

##### Anastomosis‐related complications

The results from the two studies could not be directly pooled. Noh et al. [20] reported 60 (9.6%) anastomosis‐related complications among the surgeon group with prior laparoscopic experience and one (2.9%) in the surgeon group with no prior laparoscopic experience. This might not be due to the difference in complexity of cases in the two groups as there was no significant difference in the tumour locations. However, this could potentially be because of the discrepancy in the total number of cases performed in the two groups (n = 628 in prior laparoscopic experience group versus *n* = 34 in the no prior laparoscopic experience group). In Sian et al. [21], there were no anastomotic leaks in either group.

##### Learning curve measured in terms of simulator performance

Only one study [22] contained data on the learning curve measured in terms of robotic simulator performance. This was an observational study that compared the performance of a novice in laparoscopic surgery with that of an intermediate operator (≤100 laparoscopic cases) and expert operator (>100 laparoscopic cases). It concluded that there was no significant difference in the overall simulation score among the three groups in three of the four simulation tasks. The laparoscopic novice outperformed experts in one of the tasks (*p* = 0.004). Laparoscopic intermediates did not significantly differ from the other two groups in overall simulation score. There was no pattern or difference between groups in terms of parameters of specific simulator tasks.

This study was not specific to colorectal procedures and assessed whether laparoscopic skills were transferable to robotics in general. It was hindered by a small sample size and differences in size between the experience groups (novice 41, intermediate 8, expert 11), limiting the generalizability of the findings. Furthermore, the study was observational with no randomization, blinding or allocation concealment. Another shortcoming of the study was that the intermediate and expert laparoscopic groups were potentially rather heterogeneous as they were defined by the previous number of laparoscopic procedures performed not considering the complexity of previously performed procedures.

#### Future research priorities

The current literature seems to suggest that prior laparoscopic experience may not have an effect on the learning curve of robotic colorectal surgery measured in terms of clinical outcomes. However, the literature on the topic specific to robotic colorectal surgery is scarce and of very low quality. Future research in the field is required to define prior experience and establish a learning curve by measuring performance for homogeneous case loads in comparative studies.

**Question 4:** What are the effects of having prior experience of one robotic platform (for learners) versus no such prior experience on the learning curve for learning another robotic platform?

#### Recommendation

No recommendation could be provided regarding the effects of having prior experience of one robotic platform versus no such prior experience on the learning curve for learning another robotic platform.

**Question 5:** What are the effects of simulation training (for learners) versus no simulation training on operative performance in colorectal robotic surgery?

**Question 6:** What are the effects of simulation training (for learners) versus no simulation training on patient outcomes in colorectal robotic surgery?

#### Recommendation

Simulation should be used as part of the colorectal robotic training curriculum. [Very low level of evidence and expert opinion, upgraded by the guideline working group; *strong recommendation*]

#### Rationale for recommendation

Although there is very little evidence on these topics, the guideline group felt that simulation should be offered as part of the robotic training curriculum based on the balance of benefits versus harm, as most robotic users have access to simulation and these include skill exercises and some procedural exercises. There is a scarcity of data on the effect of simulation training in colorectal robotic surgery with regard to operative performance and patient outcomes. Simulation training could potentially increase trainees' confidence and familiarity with the robotic platform. The most important reason to adopt effective simulation training is to ensure patient safety, in particular to avoid an increased rate of adverse clinical outcomes at the beginning of a surgeon's learning curve. Simulation training can potentially ensure that a trainee surgeon develops a certain level of competence in robotic skills and familiarity with the robotic platform in a safe environment before starting his or her first robotic case. Apart from simulation in basic surgical skills, a few simulation models designed specifically for robotic rectal dissection have been developed [23, 24].

A recent study has shown that although 63% of US residents indicated that they had participated in robotic cases, only 18% had experience using the robotic console and 60% received no prior education or training before their first robotic case [25]. Indeed, integrating surgical trainees into robotic procedures is challenging, especially in the initial phase of launching a new robotic service in a centre. One contributing factor is the lack of formal and mandatory robotic simulation curricula. Formal robotic platform training and simulation may allow increased trainee participation and skill acquisition in robotic procedures.

This section explores the effect of simulation training on operative performance and patient outcomes in colorectal robotic surgery. Eight articles were considered relevant to this statement [23, 24, 25, 26, 27, 28, 29, 30]. Three of the eight articles have data on operative performance and one of the eight articles has data on patient outcome.

##### Operative performance

The three studies that had data on operative performance were Schlottman et al. (2019), Thomas et al. (2023) and Cho et al. (2013) [26, 27, 28]. The data from these studies cannot be synthesized as the specific operative performances they measured were different. We felt that the three studies were not suitable for quality assessment through GRADE because GRADE is an outcome‐based assessment and the three studies did not have a common outcome measure. Specifically, Schlottman et al. [26] did not have an outcome measure of critical importance, Thomas et al. [27] is a conference abstract with limited data. The outcomes of Cho et al. [28] were specific to that study and did not conform to known outcome measures.

Schlottman et al. [26] was an observational study comparing the confidence level of 20 senior surgical residents in using the robotic platform before and after simulation training with porcine tissue blocks to perform various operations including Heller myotomy, sleeve gastrectomy, colectomy and lobectomy. It showed a significant increase in confidence level in port placement (5.36 vs. 7.05, *p* = 0.007), docking (5.59 vs. 7.18, *p* = 0.01), suturing (5.05 vs. 7.50, *p* < 0.001), using an energy device (5.36 vs. 7.36, *p* < 0.001) and using staples (4.91 vs. 7.41, *p* < 0.001) after 3 days of simulation training. The highest increases in confidence level were seen in skills often less readily developed in theatre without prior practice, such as suturing and using energy devices or staples. The limitation is that the sample size was small and that the study was not limited to colorectal robotic procedures.

Thomas et al. [27] is a conference abstract; 19 surgical residents practised during a training session using a live porcine model consisting of training components: port placement, docking, cholecystectomy, bowel resection and anastomosis. Perception of the exercise, confidence in technical skills and attitudes regarding robotic surgery were rated before and after the exercise using Likert scales. Following the exercise, all participants reported an improvement in robotic surgical ability.

Cho et al. [28] was a randomized controlled study aimed at evaluating if robotic skills acquired in a virtual environment could be applied to actual complex da Vinci procedures by comparing senior surgical residents who received virtual reality (VR) training (Group 1) and those who did not (Group 2). It was found that the VR index (a composite VR score) was significantly greater in Group 1 compared with Group 2 after Group 1 had received training (19.3 ± 4.5 vs. 9.7 ± 4.1, *p* = 0.001). Group 1 also showed improved performance in the da Vinci exercise compared with Group 2. The study was limited by no blinding and a small sample size, involving six senior surgical residents in each group. This study was not limited to robotic colorectal surgical skills as it evaluated surgical skills in general.

To date, there have been no studies that specifically examine the role of simulation training in the transfer of specific skills for performing robotic colorectal surgery.

##### Patient outcome

Zern et al. (2015) [30] is the only article identified that has data on patient outcome. It is a conference abstract that described an observational study in which eight advanced laparoscopic surgeons participated in simulation drills, and the outcomes of the first 208 general surgical procedures performed using the da Vinci Si system following simulation training were evaluated. There were no robotic‐specific complications, with a robotic to laparoscopic conversion rate of 3.4% and robotic to open conversion rate of 0.96%, compared to other surgical disciplines at the institution that started robotic surgery without simulation that had conversions and complication rates of over 8%. The study was not specific to robotic colorectal surgery. It was unclear what specific complications the study was examining, and the sample size of the comparator was not apparent. The overall sample size of this abstract is decent but it was unclear how many of them were robotic colorectal procedures. It lacked refined details, which hampered interpretation and reproducibility.

#### Future research priorities

Most of the currently available simulators are focused on basic skills training, and the effects of procedural simulation in robotic colorectal surgery are yet to be determined.

There are no comparative data examining the effect of use of simulation in operative performance and patient outcomes specifically in robotic colorectal surgery. Simulation training could potentially improve trainees' confidence, familiarity with the robotic platform and performance in skill and procedural exercises. It remains unclear whether simulation training improves clinical outcome in robotic colorectal surgery.

Research priorities in this area should include defining a core set of critical clinical outcomes, and future work should focus on exploring whether simulation training could improve these critical clinical outcomes.

**Question 7:** What are the effects of using mentoring (for learners) versus no mentoring on clinical outcomes in training colorectal robotic surgery?

#### Recommendations

In‐person intraoperative mentoring should be used for surgeons performing their initial colorectal robotic cases. [Expert opinion only]

#### Rationale for recommendation

Although there is a very low level of evidence on this research question, based on the benefits versus harms, for patient safety, in‐person intraoperative mentoring should be considered in the initial cases in robotic colorectal training. Some published papers on the modular training programme in robotic colorectal surgery incorporating in‐patient intraoperative mentoring (see Question 9 on the modular approach on procedural training) have shown that modular training is safe. Proctorship and mentoring are also recommended by the recent opinion‐based guideline published by the Royal College of Surgeons of England [31]. However, there is no direct comparison assessing the effects of using mentoring versus no mentoring on clinical outcomes in colorectal robotic surgery training.

Two papers that focused on this topic were identified. Stefanidis et al. [32] identified technical and nontechnical errors made by minimally invasive surgeons and devised a coaching programme (as opposed to mentoring/proctoring) designed to provide feedback and reduce these errors. There was no assessment of patient outcomes or technical skills postcoaching and so it is not possible to draw any conclusions about the effectiveness of the intervention. There is also a narrative review by Zahid and Miskovic [33], who outline the attributes of a successful proctor and provide advice on establishing a high‐quality proctoring programme, but do not provide any new evidence for mentoring in robotic colorectal surgery.

There is often confusion when discussing the terminology for mentoring, preceptoring, proctoring and coaching, and the definitions of these terms are summarized in the ‘Definitions and glossary’.

#### Future research priorities

Future research to assess the training for learners and patients’ clinical outcomes should be clear about the terminology, whether it is mentoring, preceptoring, proctoring or coaching. It is a common practice to have experienced robotic colorectal surgeons guide learners in the early part of the learning curve, maybe through mentoring or preceptoring. Would it be feasible or ethical to compare the effects of mentoring/preceptoring versus no mentoring/preceptoring? How are the trainees getting the support of experienced robotic colorectal surgeons? Currently, many of these training activities are supported by industry, although scientific‐led training activities have started. It would be of value to identify the type of in‐person training (e.g. mentoring/preceptoring) used, methods employed, duration and frequency and the effect on learners and patient outcomes.

**Question 8:** What are the effects of telementoring versus onsite mentoring (for learners) on clinical outcomes in colorectal robotic surgery?

#### Recommendation

Telementoring could be considered to support mentoring in training colorectal robotic surgery in selected cases. [Very low level of evidence; *conditional recommendation*]

#### Rationale for recommendation

There were no comparative studies comparing the effects of telementoring versus onsite mentoring on clinical outcomes in colorectal robotic surgery.

Augestad and colleagues [34] carried out a literature review on the clinical outcomes and educational benefits of telementoring in surgery, and found that there is very limited research, often with conflicting outcomes. Participant satisfaction with telementoring programmes was found to be high, but this did not appear to translate into improved clinical outcomes or knowledge transfer. They recommend future research focused on both these areas with respect to telementoring.

Butt et al. [35] have shown that telementoring can be used within existing operating theatre set‐ups as an adjunct to a structured educational programme.

Sebajang et al. [36] described a series of 18 laparoscopic colorectal cases that were either telementored or telerobotically assisted (where the remote expert surgeon is in control of a robotic arm, mainly used for assistance). They proposed that both methods could be used to overcome the learning curve of minimally invasive colorectal surgery, but had no comparator group.

It is felt that the studies by Augestad et al., Butt et al. and Sebajang et al. were not suitable for quality assessment through GRADE because they did not have a common outcome measure. Therefore, GRADE assessment was not performed.

These studies suggest that telementoring may have a role in shortening the learning curve and can be useful for surgeons at the beginning of their robotic experience, but there is a very low level of evidence that telementoring should be included in any colorectal robotic surgery curriculum. Due to the heterogeneity in measured outcomes between these three papers no firm conclusion can be drawn in terms of effectiveness of this outcome. Augestad et al. commented on the lack of standardized terminology in this area, with the consequence that meaningful comparison between studies is difficult.

#### Future research priorities

Further research is required to assess how learners attain proficiency in surgical techniques between telementoring and onsite mentoring and their effects on clinical outcomes in colorectal robotic surgery, particularly as the availability of expertise online has the potential to make training more accessible.

**Question 9:** What are the effects of the modular approach on procedural training in the operating room (for learners) versus not using the modular approach on the learning curve of colorectal robotic surgery training?

#### Recommendation

A modular approach to procedural training in the operating room could be considered in robotic colorectal surgery training. [Very low level of evidence and expert opinion; *conditional recommendation*]

#### Rationale for recommendation

Twenty articles were considered relevant to this research question [37, 38, 39, 40, 41, 42, 43, 44, 45, 46, 47, 48, 49, 50, 51, 52, 53, 54, 55, 56]: three were comparative studies [37, 38, 39], 11 were case series [40, 41, 42, 43, 44, 45, 46, 47, 48, 49, 50] and six were consensus statements, review articles and an Editorial [51, 52, 53, 54, 55, 56].

##### Safety of adopting modular training

Three comparative articles [37, 38, 39] demonstrate the safety of adopting modular training. Panteleimonitis (2021) [37] included the data from their 2018 study [38], and therefore only the 2021 study is discussed below. The results from Panteleimonitis [37] and Simpson (2022) could not be pooled as the work by Simpson et al. (2022) [39] is an abstract and did not provide a denominator for their outcomes.

Panteleimonitis et al. [37] was a multicentre observational study that compared the short‐term patient outcomes of robotic colorectal surgeries performed by surgeons who, at the time of the study, were being trained under the European Academy of Robotic Colorectal Surgery (EARCS), which provided a framework, guidelines and modular approach in training and assessment, with that of EARCS graduates (surgeons who had finished training with EARCS) and proctors. EARCS was formed by a group of robotic expert surgeons and is not a scientific society.

There were 323 EARCS trainee cases, 626 EARCS graduate cases and 181 EARCS proctor cases. Across the three groups, there was a statistically significant difference in terms of length of stay (7 vs. 6 vs. 6 days, *p* = 0.003), operating time (302 vs. 265 vs. 255 min, *p* < 0.001) and estimated blood loss (50 vs. 50 vs. 30 mL, *p* < 0.001). However, there was no significant difference in conversion rate (2.2% vs. 3.4% vs. 2.8%, *p* = 0.583), 30‐day reoperation (6.5% vs. 6.2% vs. 5.5%, *p* = 0.908), 30‐day readmission (7.1% vs. 8.1% vs. 8.3%, *p* = 0.835), 30‐day mortality (0.3% vs. 0.3% vs. 0%, *p* = 0.750), anastomotic leak (3.1% vs. 3.2% vs. 3.3%, *p* = 0.954), Clavien–Dindo complications (*p* = 0.714), R1 resections (1.5% vs. 1.7% vs. 2.3%, *p* = 0.863) or lymph node yield (18 vs. 18 vs. 18, *p* = 0.778).

The limitation of Panteleimonitis et al. [37] is that it might have suffered from selection bias during the process of choosing patients for proctorship and training. The data were collected prospectively but were surgeon‐reported and there was a risk of reporting bias and inadequate data entry. Also, it was a study that involved 26 different centres. Each centre might have had different criteria for patient selection and it might have been difficult to control confounding factors such as postoperative care and discharge criteria.

Simpson et al. [39] was a single‐centre observational study that compared the clinical outcomes of robotic total mesorectal excision (TME) for rectal cancer in which steps were performed by surgical trainees trained in a modular fashion under supervision, with those of robotic TME performed entirely by consultant surgeons. It was a conference abstract, and it was unclear how many patients were included in the study. They showed that there was no significant difference in clinical (morbidity rates, intraoperative blood loss, operating time and length of stay) and oncological outcomes (R0 resection, lymph node count, 5‐year overall survival) between the two groups. Fewer than 5% of patients had an anastomotic leak/return to theatre.

Simpson et al. [39] was limited by the fact that it was a conference abstract and therefore lacked details. It was unclear how patients were selected and if there was any selection bias. It was unclear how many patients were included in the study. The methodology was also unclear in terms of the extent of trainee involvement in the cases.

#### Future research priorities

There were no data in the literature comparing the learning curve of robotic surgery trainees who were trained in a modular fashion with those who were not. However, literature shows that a modular training approach is safe. Further studies are required to establish whether a modular approach could shorten the training required to achieve proficiency in robotic colorectal surgery. Significant postoperative complications are infrequent, and it is perhaps worth setting minimally important differences in future studies to assess this.

**Question 10:** What are the effects of attending a structured TTT course for robotic surgery (for the trainer) versus not attending such a course on the operative performance (of the trainee) of colorectal robotic surgery training?

#### Recommendation

A structured TTT course could be considered for robotic colorectal surgery. [Very low level of evidence and based on expert opinion; *conditional recommendation*]

#### Rationale for recommendation

Essentially there are only consensus and expert opinions to support trainers undergoing specific TTT courses for robotic surgery. The current evidence lacks reporting of its impact on clinical outcomes and its effectiveness compared with generic TTT course training.

In 2019, Gómez Ruiz et al. published an expert consensus on a TTT curriculum for robotic colorectal surgery, which was accompanied by a published editorial [57, 58]. Fourteen experts in robotic surgery (defined as having performed a minimum of 50 robotic colorectal resections and being involved in training in colorectal surgery) participated using a modified Delphi process. All participants were male. All agreed that there is a need for a TTT course in robotic surgery that should include the following components with a modular approach: evidence for robotic surgery, nontechnical skills, patient safety, critical review of teaching and training methods and formal assessment prior to completion. Participants also reported value in gaining advice on working with a ‘difficult trainee’, how to optimize feedback and the practical teaching of technical skills. There was no consensus on the role of virtual reality but the authors recognized that this may change as technology advances.

The effectiveness of a robotic TTT course was described by a group of expert surgeons and trainees in 2020, Eardley et al. [59] published the results of a pilot experience of robotic TTT on behalf of the European School of Coloproctology of the ESCP. In this study, a pilot TTT course was devised from evidence from a scoping review by an experienced robotic surgeon and gastroenterologists with vast TTT course experience. Eight delegates participated in the course with a 2:1 delegate:faculty ratio. Delegate feedback was positive across several domains with an open dialogue and good balance of theoretical learning and practical exercises reported. In particular, delegates reported a benefit in devising a ‘common language’ for the description of intraoperative movements in the training process. The impact on operative performance was not directly assessed.

#### Future research priorities

The requirement for a specific robotic surgery TTT course requires further investigations, particularly in regard to how such a course compares in effectiveness with a generic TTT course, and the measure of effectiveness should include clinical outcomes.

**Question 11:** What are the effects of nontechnical skills training (for learners) versus no nontechnical skills training on patient safety in colorectal robotic surgery?

#### Recommendation

Nontechnical skills training could be considered as part of a colorectal training curriculum. [Very low level of evidence; *conditional recommendation*]

#### Rationale for recommendation

Three papers were identified on this topic. AlJamal et al. [60] conducted a small‐scale pilot assessment of a simulation‐based nontechnical skills curriculum for robotic surgery, evaluating six colorectal surgery residents. Participants were observed during two simulated scenarios and provided self‐ratings using the Interpersonal and Cognitive Assessment for Robotic Surgery (ICARS) tool. Expert faculty also rated each participant and provided personalized feedback after each case. Scenarios were then repeated 6 months later, with repeat ICARS ratings. Significant improvements were demonstrated in the fields of leadership, decision‐making and situational awareness. This study was deemed to be of low quality (due to its observational nature) but was not further downgraded.

El‐Hamamsy et al. [61] reported an observational study and qualitative interviews of team communication in robotic surgery. They hypothesized that robotic surgery provides unique communication challenges due to surgeon–team separation and suggested solutions including staff training and team consistency. They acknowledged that further studies are needed on this topic. These findings are echoed in an integrative literature review by Mathew and colleagues [62], where intraoperative communication, teamwork and disruptions were identified as the three key factors affecting patient safety during minimally invasive surgery.

There appears to be an acceptance in the literature [60, 61, 62] that robotic surgery can lead to communication issues (and therefore nontechnical skills training is likely to be useful) but there are low levels of evidence to support mandatory inclusion of nontechnical skills training in a colorectal robotic surgery curriculum.

#### Future research priorities

Studies could examine the objective outcomes between programmes with nontechnical skills training versus those without and the effects on patient safety.

**Question 12:** What are the effects of competency‐, proficiency‐based supervised training (for learners) versus noncompetency‐, nonproficiency‐based supervised training during colorectal robotic surgery training on operative performance?

**Question 13:** What are the effects of competency‐, proficiency‐based supervised training (for learners) versus noncompetency‐, nonproficiency‐based supervised training during colorectal robotic surgery training on patient clinical outcomes?

#### Recommendation

No recommendation could be provided on the effects of competency‐, proficiency‐based supervised training (for learners) versus noncompetency‐, nonproficiency‐based supervised training during colorectal robotic surgery training on operative performance and patient clinical outcomes.

#### Rationale for recommendation

While competency‐ or proficiency‐based supervised training is well established as an effective method of skill acquisition in surgery and minimally invasive surgery, to date there are limited data on the specific realm of robotic colorectal surgery [63].

While six studies were identified as potentially relevant to the above statement, most focused on generating assessment tools with a group of expert robotic surgeons, with no reference to the specific impact on operative performance or patient clinical outcome. As they were not comparable, a summary of individual evidence is provided.

Petz et al. [64], in an expert consensus, summarized recommendations for structured training and competence assessment in robotic colorectal surgery. The methodology involved a round‐table discussion at the 6th Clinical Robotic Surgery Association (CRSA) congress using a nominal group technique. The authors summarized areas requiring competency based on general robotic (docking, trocar positioning, device training) and specific robotic colorectal (tissue handling, vessel dissection, intracorporeal anastomosis, bowel resection) skills. The impact of competency‐ or proficiency‐based supervised training on clinical outcomes in robotic colorectal surgery was not referenced.

Tou et al. [65], in a European expert consensus, reported on the assessment steps that should be included when assessing the training performance of robotic‐assisted low anterior resection. Rather than subjective assessment using a Likert‐scale scoring, a binary metric‐based proficiency‐based progression (PBP) performance assessment was used. The metrics were developed via different stages and validated using a modified Delphi technique with a group of expert robotic surgeons (face and content validity); a follow‐up study obtained a construct validity of the metrics, i.e. the developed metrics were able to different between expert versus novice surgeons [66]. However, no data on colorectal robotic surgery training with the PBP assessment method on patient clinical outcomes are available.

Haig et al. [67], in an observational study, reported on the initial experience, specifically related to usability, of the Versius robotic surgery system (CMR Surgical, Cambridge, UK). Seventeen surgical teams participated and the outcomes focused on identifying areas to improve use of the surgical system for future development. No operative or clinical outcomes were assessed or reported and neither competency‐ nor proficiency‐based supervised training was assessed.

McNair et al. [68], utilizing COSMIN (COnsensus‐based Standards for the selection of health Measurement INstruments) methodology, critically appraised assessment tools for analysing surgeon experience of novel invasive procedures and devices. They identified the Surgery Task Load Index (SURG‐TXL) to be the most valid and suitable instrument for measuring surgeon experience in innovative procedures and devices. Neither competency‐ nor proficiency‐based supervised training was assessed.

Louridas et al. [69], in a narrative review, summarized available literature on competency‐based education in both minimally invasive and robotic colorectal surgery. The authors focused this review on understanding learning curves, choice of assessment tools, standard setting and potential transferability of competency in laparoscopic surgery to robotic surgery.

Schmidt et al. [70], reported on the development and validation of an objective, structured assessment tool specific to minimally invasive (laparoscopic or robotic) linear‐stapled, hand‐sewn intestinal anastomosis (the A‐OSATS score; Anastomosis‐Objective Structured Assessment of Technical Skills). The authors recommended that the ability of the A‐OSATS tool to predict capabilities of patient outcome required further evaluation. Neither competency‐ nor proficiency‐based supervised training was assessed.

#### Future research priorities

The training curriculum now focuses more on standard‐based training, i.e. competency‐, proficiency‐based rather than training defined by a specific duration or number of cases performed. One of the barriers to implementing standard‐based training is defining a suitable assessment methodology. Currently, there are several assessment methods in robotic colorectal training, and most focus on basic skills rather than an entire procedure. It would be crucial to identify a robust method for procedural assessment. Some assessments have achieved validation, but the correlation of operative performance or patient clinical outcomes is lacking and requires further research.

**Question 14:** What are the effects of credentialing (for the practitioner) versus no credentialing in colorectal robotic surgery on patient clinical outcomes?

#### Recommendation

Credentialing could be considered for defining requirements in commencement and maintenance of robotic colorectal surgical practice. [Very low level of evidence and based on expert opinion; *conditional recommendation*]

#### Rationale for recommendation

There are no defined guidelines on proficiency required for individual practice or case load and time interval between cases for maintenance of practice [71]. In published data, many individual hospitals rely on the number of proctored cases as a surrogate for proficiency [72]. Expert consensus based on a Delphi study recommends that a credentialing process should be put in place and includes recommendations for both initial credentialing requirements and maintenance of robotic privileges such as annual volume, complication rates, return to the operating room and readmission rates [72]. Additional recommendations made by the panel included the use of procedural video review and objective assessments as metrics for assessment of proficiency, maintaining databases for outcomes and using simulation where performance concerns arise [72].

#### Future research priorities

Credentialing is not confined to robotic surgery; it is more commonly practised in the United States than in European countries. A consensus statement has supported the credentialing process in robotic surgery to ensure the proficiency of surgeons who perform robotic surgery. Though we do not have data linking the credentialing process to a surgeon's performance and patient outcomes, these would be much‐needed data. The credentialing process deals with the initial practice of robotic surgery for trained consultants and trainees who have performed robotic fellowship and their ongoing practice. The types of credentialing criteria were also suggested in the consensus statements, such as video performance of the operative surgeons and patient outcomes, and these would require validation research and resources to support further studies in these areas.

**Question 15:** What are the effects of registering clinical outcome data (for the practitioner) versus no registering in colorectal robotic surgery on patient clinical outcomes?

#### Recommendation

Registering clinical outcome data could be considered in a structured colorectal robotic training curriculum. [Expert opinion only]

#### Rationale for recommendation

We do not have any evidence of the effects of registering clinical outcome data in colorectal robotic surgery on patient outcomes in a training curriculum, although unless it is measured, it is difficult to ascertain the impact on patient safety during surgeons' training in robotic colorectal surgery. Setting up a registry would require human and financial resources and has no additional impact on patient safety, and therefore should be supported. In recent studies [71, 72], a group of experts on robotic surgery did support maintaining a database of clinical outcomes to provide evidence and guide practice. The importance of outcome auditing has been widely practised and therefore is not confined to robotic surgery.

#### Future research priorities

The database should have the following information: surgeon levels, prerobotic surgery experience, colorectal training methods, duration and assessment types, clinical data, including types of robotic colorectal procedures, preoperative demographic details, intraoperative complications, postoperative clinical outcomes including complications, mortality, length of hospital stay, histology outcomes and functional outcomes. The establishment and management of the database registry should be independent and free from the influence of robotic platform manufacturers.

## DISCUSSION

Since the first robotic colorectal procedure was performed in 2001, the number of robotic colorectal operations has increased steadily [1, 73]. Industry has led training in robotic procedures, but structured training in robotic colorectal surgery was conducted by a scientific society until the ESCP [5, 74]. When surgeons are learning new techniques patients should not come to harm, and the working group of this guideline came together to produce up‐to‐date available evidence relating to training in robotic colorectal surgery to provide guidance. This guidance is much needed, particularly when more robotic platforms are entering the market.

The working group decided there were several categories of research questions (PICOs) that are important in the robotic guideline curriculum, and these were categorized into six domains: (1) the required knowledge, (2) technical skills, (3) nontechnical skills, (4) assessment of competency/proficiency during training, (5) credentialing and (6) clinical outcome data registry in robotic colorectal surgery. From these six domains, recommended statements were formed by the guidelines working group.

Although no studies have examined the outcomes between trainees who did or did not have platform training (basic technology training), the working group felt this was essential as this is for the safety of patients. In terms of gaining the required knowledge for robotic colorectal training, no study compared eLearning with traditional learning. Increased knowledge is acquired through the internet and websites such as AIS Channel [75] and YouTube. Much of this content is unregulated, and dedicated teaching materials, whether traditional, eLearning or blended learning, with clear aims, objectives and assessment components for educational purposes are needed.

When robotic surgery started, it was thought that surgeons with prior laparoscopic skills were desirable before being considered for robotic colorectal training [64]. Two comparative studies were included in this guideline. They suggested that previous experience in laparoscopic surgery is not essential for training in robotic colorectal surgery, although the level of evidence was low. Given that increasingly more robotic platforms are coming to the market, the guideline group also looked at the effect of training in one robotic platform on training in another. Still, there were no studies that examined this aspect. Future studies could examine the impact of skill transfer for learning robotic colorectal surgery between different robotic platforms.

Training in surgery is evolving, and efforts should be made to acquire the necessary skills outside the operating room to minimize patient risk during the surgeon's learning curve. Simulation using different models, for example simulation software, dry‐lab and wet‐lab, can provide essential skills for surgeons before embarking on treating patients. Most of the currently available simulations are focused on basic surgical skills, and procedural simulation is emerging. It would be interesting to assess the effects on the learning curve and patient safety when procedural simulation is introduced. Many included case series have shown the acquisition of skills using simulation, case observation, dry‐lab and wet‐lab, and mentored cases and have shown safe implementation of robotic colorectal surgery programmes. However, there has been no direct comparison between surgeons trained through these programmes and surgeons who did not go through these programmes or use components of these adjuncts.

Modular teaching in colorectal surgery was popularized during skill acquisition in the early adoption of laparoscopic surgery [7]. Modular learning is not confined to surgery and is used in academia to facilitate learning [76, 77]. It was shown to be a structured and effective method in procedural training in urology [78]. Panteleimonitis and coworkers looked at more than a thousand robotic colorectal cases and demonstrated the safe adoption of robotic surgery using a modular training approach [37].

Emphasis has also been placed on training for the trainers when introducing robotic colorectal surgical training. The TTT course was widely adopted in the United Kingdom when the standards of endoscopic procedures required improvement [79, 80] and was later adopted in laparoscopic colorectal training with good effects [81]. An expert consensus was described in the robotic TTT curriculum by Gómez Ruiz and coworkers about the optimal components for the course [57]. A year later, in 2020, Eardley and colleagues described the pilot experience of the ESCP Colorectal Robotic Surgery Training for the Trainers course and found that delegates increased their knowledge of each course's learning objectives and identified learning points to change their practice. The feedback from the delegates on the course was positive [59].

Technologies have evolved and hold the potential to support the apprenticeship model with video‐based teaching, which in turn has aided the development of telesurgery and telementoring [82]. Telementoring allows expert surgeons to teach surgical colleagues or trainees in real time from a distance. It is feasible and as effective as onsite mentoring [83]. Despite some promising developments, no comparative study has compared the effects of telementoring versus onsite mentoring on clinical outcomes in colorectal robotic surgery.

Increasing focus on patient outcomes is placed not only on the knowledge and skills of the surgeons but also on nontechnical skills. Evidence has shown that many adverse surgical events resulted from behavioural or nontechnical aspects of performance rather than a lack of technical expertise alone [84]. Nontechnical skills such as team working, leadership, situation awareness, decision‐making and communication are paramount in the safety and outcomes of patients [85]. As the complexity of the theatre set‐up for robotic surgery has increased the distance between the surgeon and the patient, assistant, scrub nurse and anaesthetist, more significant demands are placed on nontechnical components such as communication and situation awareness. There was low‐level evidence supporting the mandatory inclusion of nontechnical skills for surgeons training in a colorectal robotic surgery curriculum.

Many factors influence patient outcomes, and since a landmark paper published by Luft and colleagues in 1979 [86], the volume–outcome relationship has been utilized by surgical societies and organizations to synonymize the volume of operation with competency/proficiency. However, confidence in the volume and patient outcome relationship has been challenged after a seminal study demonstrated the skill–outcome relationship [87]. Since then, several studies have reported similar findings [88, 89, 90, 91]. More emphasis is now placed on assessing competency/proficiency of knowledge/skills rather than duration or volume of practice. The studies reviewed in this guidance focused on the generation of assessment tools but gave no data on the impact of operative performance on patient outcomes in robotic colorectal surgery.

At present, there are no agreed credentialing criteria for robotic colorectal practice. A recent expert consensus from a Delphi study recommended that a credentialing process should be put in place and included recommendations for both initial credentialing requirements and maintenance of robotic privileges, such as annual volume and complication rates [72].

No study examined the effects of registering clinical outcome data versus not registering in colorectal robotic surgery on patient clinical outcomes. Despite the lack of Level 1 evidence supporting the use of robotic colorectal surgery, colorectal procedures have steadily increased over the last few years [1]. To evaluate the effectiveness of an intervention, it is crucial to assess this with the IDEAL framework and use either a RCT or registry‐type database to assess long‐term outcomes and evaluate the effectiveness of the innovation [92].

There are some limitations of the current review. The search strategy for the training was limited to robotic colorectal surgery articles. Therefore, information on robotic surgery in other specialities and laparoscopic colorectal training was not assessed. As more data emerge relating to robotic surgery, we may be in a better position to assess the transferability of established training and teaching techniques and assessment tools [93] to robotic colorectal surgery.

Despite a good uptake in robotic colorectal surgery, training in robotic surgery is at very early stages and has mainly been industry‐led. Many research questions in this guideline had little or no good quality comparative data. There were primarily case series, and these studies did not assess many predefined outcomes.

When providing the recommendations, the working group had to determine the quality of evidence for the research questions and the balance between benefits and harms. Many of the current training practices are perceived to benefit patient safety and reduce the learning curve. Still, better evidence is needed to assess the effectiveness of these training interventions.

To facilitate the implementation of the recommendations from this guideline, we will, once published, announce them in social media via ESCP social feeds, the ESCP Guidelines website and the ESCP Colorectal Robotic Surgery Working Group website and robotic webinars; these websites and webinars are often viewed by expert robotic trainers, trainees, opinion leaders and industry partners. We will encourage the trainers/trainees to share the guidelines with their hospital theatre team and leadership.

The cost of robotic surgical training is considerable [94], and most articles relating to robotic training used for this guideline were published in developed countries. Policymakers and hospital practitioners would find some recommendations in this guideline helpful for low‐ to middle‐income countries when implementing robotic colorectal training.

### Research gaps

Research gaps for each research question were mentioned under each section. However, there are some prioritized items for which research is needed. The outcome measure of training appears to be limited to what could be measured as skill‐specific time or completeness of task. Whether these factors, for example operative time and conversion rate, translate to improved patient clinical outcomes has not been well studied. Many of the proposed training interventions were to minimize the learning curve when surgeons embark on robotic colorectal training. Improving surgical performance over time is described as the learning curve [95]. Cook and coworkers proposed three features of the learning curve: (1) the initial level of performance, (2) the learning rate, i.e. how quickly performance improves, and (3) a plateau that represents the stabilization of performance [96]. Surrogate markers such as operating time, blood loss and patient outcomes are often used as a proxy for learning, as surgical skills may not be easily quantified [97].

## CONCLUSION

This guideline provides the best evidence available regarding the robotic colorectal training curriculum. While the evidence is lacking in some areas, the working group assessed each component of training with available evidence and considered harm versus benefits for each training intervention. The working group has also identified priorities for future research. This document serves as guidance, and the curriculum will adapt as more evidence becomes available in the future.

## REVIEW AND QUALITY ASSURANCE

This guideline was presented at the ESCP annual scientific conference in September 2022 in Dublin; comments were incorporated in the revision of the manuscript. The ESCP Guidelines and Research Committee members Stephanie Breukink and Gabrielle van Ramshorst provided constructive reviews of this guideline's content and methodology. This guideline went through the peer review during the submission process in Colorectal Disease.

## AUTHOR CONTRIBUTIONS

**Samson Tou:** Conceptualization; investigation; funding acquisition; writing – original draft; methodology; writing – review and editing; supervision; visualization; project administration; data curation; formal analysis. **Stephanie Au:** Writing – original draft; methodology; writing – review and editing; formal analysis; data curation; investigation; visualization. **Cillian Clancy:** Writing – original draft; methodology; writing – review and editing; data curation; investigation. **Steven Clarke:** Writing – review and editing. **Danielle Collins:** Conceptualization; writing – review and editing; supervision. **Frances Dixon:** Writing – original draft; methodology; writing – review and editing; data curation; formal analysis; investigation; visualization. **Elizabeth Dreher:** Writing – review and editing. **Christina Fleming:** Writing – original draft; methodology; writing – review and editing; formal analysis; data curation; resources; investigation; project administration. **Anthony G. Gallagher:** Writing – review and editing. **Marcos Gomez‐Ruiz:** Writing – review and editing; conceptualization; funding acquisition. **Jos Kleijnen:** Methodology; conceptualization; resources; writing – review and editing. **Yasuko Maeda:** Conceptualization; writing – original draft; methodology; writing – review and editing; supervision; visualization; resources. **Katie Rollins:** Data curation; writing – review and editing. **Klaus E. Matzel:** Supervision; writing – review and editing; funding acquisition; conceptualization; resources.

## CONFLICT OF INTEREST STATEMENT

ST received education grants from Intuitive Surgical and Medtronic. MGR received education grants from Intuitive Surgical and Medtronic and is currently a medical advisor to Intuitive Surgical, Medtronic and J&J. AGG holds education research grants from Medtronic (Dublin, Ireland) and the Arthroscopic Association of North America (Chicago, USA) for the investigation of metric‐based education and training. KEM is medical advisor to Medtronic and Boehringer Ingelheim. We did not receive any funding from the industry for this guideline, and the industry did not influence its conception, selection of the experts, design and conduct of the research, data collection, analysis or preparation of this guideline.

## FUNDING INFORMATION

The European School of Coloproctology of the ESCP provided the funding to commission an independent strategic literature search by Kleijnen Systematic Reviews (KSR) Ltd.

## ETHICS STATEMENT AND INFORMED CONSENT

This project did not require approval by the ethics committee.

## DEFINITIONS AND GLOSSARY

**Blended learning:** a combination of traditional learning and eLearning.

**Credentialing:** a formal process that provides formal accreditation of competencies/proficiencies (skills, knowledge).

**Coaching:** coaching aims to develop various skills for learners (coachees). Coaching involves a collaborative relationship between the coach and the coachee, and the coach and the coachee jointly establish individualized goals and objectives.

**Competency:** competency represents the minimal threshold for skill development.

**Effectiveness of teaching:** defined as shortening the learning time to achieve the same amount of knowledge/skills or acquiring/retaining more knowledge within the same amount of time.

**eLearning:** structured learning conducted via electronic media, typically on the internet.

**Learner:** a person learning a subject or skill.

**Learning curve:** the improvement in surgical performance over time is described as the learning curve. Cook and coworkers [96] proposed three features of the learning curve: (1) the initial level of performance, (2) the learning rate, i.e. how quickly performance improves, and (3) a plateau that represents performance stabilization. Surrogate markers for learning such as operating time, blood loss and patient outcomes are often used as a proxy for learning as surgical skills may not be easily quantified.

**Mentoring:** an intense, multidimensional professional relationship between mentor and the mentee that extends over a prolonged period and aims to develop mentees' various skills using different educational constructs.

**Modular training:** a training system whereby the trainee undertakes specific parts of the operation in a planned and structured way.

**Practitioner:** a person actively engaged in a discipline or profession, especially medicine.

**Preceptoring:** a defined and focused interaction between the preceptor and preceptee (trainee) that is especially valuable to the preceptee in acquiring practical knowledge and skills. Preceptoring may be conducted in various clinical settings in actual and simulated environments. It may be aimed at small groups or individuals.

**Proctoring:** performing summative assessments of a learner's skills using robust assessment methods. The proctor's goal is to assess the learner's performance.

**Proficiency:** a high degree of skills, expertise.

**Simulation:** any activity that aims to imitate an environment in order to inform, modify or assess surgical skills and behaviour. It could be carried out via different platforms such as virtual reality simulators, animal tissue simulators, cadavers or live animals.

**Teacher:** a person who helps others to acquire knowledge, competencies or values.

**Telementoring:** remote mentoring using telecommunication technology.

**Teleproctoring:** remote proctoring using telecommunication technology.

**Traditional learning:** face‐to‐face learning interactions that occur in a physical location, such as a lecture theatre or classroom.

## Data Availability

The data that support the findings of this study are available from the corresponding author upon reasonable request.

## SEARCH STRATEGIES

Embase (Ovid): 1974 to 3 May 2022

Date searched: 4 May 2022

Records found: 978

**Table:**

1	exp colorectal surgery/ or exp colon surgery/ or exp rectum surgery/	109240
2	exp large intestine disease/su [Surgery]	111934
3	((colorect* or rectal* or rectum* or mesorectal or colon* or sigma* or sigmo* or rectosigm* or bowel* or anal or anus or anorectal or c?ecum or c?ecal or il?eoc?ecal or il?eoc?ecum or duodenum or intestin*) adj4 (surg* or laparoscop* or keyhole or key hole or minimally invasive or MIS or resect* or excis* or dissect*)).ti,ab.	126171
4	((colitis or crohn* or diverticulitis or enterocolitis or diverticulosis or proctalgia or proctocolitis or rectocele or fistula* or fissure* or hernia* or h?emorrhoid*) adj4 (surg* or laparoscop* or keyhole or key hole or "minimal* invasive" or MIS or resect* or excis* or dissect*)).ti,ab.	42655
5	(hemicolectom* or colectom* or rectopex* or sigmoidectom*).ti,ab.	31837
6	or/1‐5	274089
7	exp robotics/ or exp robot assisted surgery/	60269
8	(robot* or robosurg*).ti,ab,hw.	100214
9	(da vinci or versius).ti,ab,hw.	6772
10	or/7‐9	100900
11	exp medical education/	346037
12	(train* or learn* or elearn* or educat* or simulat* or mentor* or proctor*).ti,ab.	2618579
13	((skill* or abilit* or knowledge or competenc* or proficienc*) adj3 (assess* or monitor* or exam* or credential*)).ti,ab.	121500
14	or/11‐13	2849030
15	6 and 10 and 14	978

MEDLINE and Epub Ahead of Print, In‐Process, In‐Data‐Review and Other Non‐Indexed Citations and Daily (Ovid): 1946 to 3 May 2022

Date searched: 4 May 2022

Records found: 429

**Table:**

1	exp Colorectal Surgery/	4105
2	exp Intestinal Diseases/su [Surgery]	134393
3	((colorect* or rectal* or rectum* or mesorectal or colon* or sigma* or sigmo* or rectosigm* or bowel* or anal or anus or anorectal or c?ecum or c?ecal or il?eoc?ecal or il?eoc?ecum or duodenum or intestin*) adj4 (surg* or laparoscop* or keyhole or key hole or minimally invasive or MIS or resect* or excis* or dissect*)).ti,ab.	82606
4	((colitis or crohn* or diverticulitis or enterocolitis or diverticulosis or proctalgia or proctocolitis or rectocele or fistula* or fissure* or hernia* or h?emorrhoid*) adj4 (surg* or laparoscop* or keyhole or key hole or "minimal* invasive" or MIS or resect* or excis* or dissect*)).ti,ab.	29939
5	(hemicolectom* or colectom* or rectopex* or sigmoidectom*).ti,ab.	19074
6	or/1‐5	210053
7	Robotics/	24545
8	Robotic Surgical Procedures/	13130
9	(robot* or robosurg*).ti,ab,hw.	63700
10	(da vinci or versius).ti,ab,hw.	3535
11	or/7‐10	64267
12	exp Education, Medical/	179347
13	(train* or learn* or elearn* or educat* or simulat* or mentor* or proctor*).ti,ab.	2132729
14	((skill* or abilit* or knowledge or competenc* or proficienc*) adj3 (assess* or monitor* or exam* or credential*)).ti,ab.	90318
15	or/12‐14	2261309
16	6 and 11 and 15	429

CINAHL (EBSCO): 1981–4 May 2022

Date searched: 4 May 2022

Records found: 104

**Table:**

S1	TI (colorect* or rectal* or rectum* or mesorectal or colon* or sigma* or sigmo* or rectosigm* or bowel* or anal or anus or anorectal or c#ecum or c#ecal or il#eoc#ecal or il#eoc#ecum or duodenum or intestin*) n4 (surg* or laparoscop* or keyhole or key hole or minimally invasive or MIS or resect* or excis* or dissect*) OR AB (colorect* or rectal* or rectum* or mesorectal or colon* or sigma* or sigmo* or rectosigm* or bowel* or anal or anus or anorectal or c#ecum or c#ecal or il#eoc#ecal or il#eoc#ecum or duodenum or intestin*) n4 (surg* or laparoscop* or keyhole or key hole or minimally invasive or MIS or resect* or excis* or dissect*)	15,489
S2	TI (colitis or crohn* or diverticulitis or enterocolitis or diverticulosis or proctalgia or proctocolitis or rectocele or fistula* or fissure* or hernia* or h#emorrhoid*) n4 (surg* or laparoscop* or keyhole or key hole or "minimal* invasive" or MIS or resect* or excis* or dissect*) OR AB (colitis or crohn* or diverticulitis or enterocolitis or diverticulosis or proctalgia or proctocolitis or rectocele or fistula* or fissure* or hernia* or h#emorrhoid*) n4 (surg* or laparoscop* or keyhole or key hole or "minimal* invasive" or MIS or resect* or excis* or dissect*)	7,054
S3	TI ( hemicolectom* or colectom* or rectopex* or sigmoidectom* ) OR AB ( hemicolectom* or colectom* or rectopex* or sigmoidectom* )	2,824
S4	S1 OR S2 OR S3	23,348
S5	(MH "Robotics+") OR (MH "Robotic Surgical Procedures")	12,053
S6	TI ( robot* or robosurg* ) OR AB ( robot* or robosurg* )	14,702
S7	TI ( da vinci or versius ) OR AB ( da vinci or versius )	742
S8	S5 OR S6 OR S7	17,663
S9	(MH "Education, Medical+")	41,623
S10	TI ( train* or learn* or elearn* or educat* or simulat* or mentor* or proctor* ) OR AB ( train* or learn* or elearn* or educat* or simulat* or mentor* or proctor* )	794,753
S11	TI ( (skill* or abilit* or knowledge or competenc* or proficienc*) n3 (assess* or monitor* or exam* or credential*) ) OR AB ( (skill* or abilit* or knowledge or competenc* or proficienc*) n3 (assess* or monitor* or exam* or credential*) )	41,347
S12	S9 OR S10 OR S11	830,397
S13	S4 AND S8 AND S12	104

Cochrane Library (Wiley): Issue 4 of 12, April 2022

Date searched: 4 May 2022

Records found:

Cochrane Database of Systematic Reviews (CDSR): 0

Cochrane Central Register of Controlled Trials (CENTRAL): 42

**Table:**

#1	MeSH descriptor: [Colorectal Surgery] explode all trees	225
#2	MeSH descriptor: [Intestinal Diseases] explode all trees and with qualifier(s): [surgery ‐ SU]	4090
#3	((colorect* or rectal* or rectum* or mesorectal or colon* or sigma* or sigmo* or rectosigm* or bowel* or anal or anus or anorectal or c?ecum or c?ecal or il?eoc?ecal or il?eoc?ecum or duodenum or intestin*) near/4 (surg* or laparoscop* or keyhole or key hole or minimally invasive or MIS or resect* or excis* or dissect*)):ti,ab	11196
#4	((colitis or crohn* or diverticulitis or enterocolitis or diverticulosis or proctalgia or proctocolitis or rectocele or fistula* or fissure* or hernia* or h?emorrhoid*) near/4 (surg* or laparoscop* or keyhole or key hole or "minimal* invasive" or MIS or resect* or excis* or dissect*)):ti,ab	3575
#5	(hemicolectom* or colectom* or rectopex* or sigmoidectom*):ti,ab	1448
#6	#1 or #2 or #3 or #4 or #5	16981
#7	MeSH descriptor: [Robotics] this term only	719
#8	MeSH descriptor: [Robotic Surgical Procedures] this term only	388
#9	(robot* or robosurg*):ti,ab	5664
#10	(da vinci or versius):ti,ab	405
#11	#7 or #8 or #9 or #10	5749
#12	MeSH descriptor: [Education, Medical] explode all trees	3541
#13	(train* or learn* or elearn* or educat* or simulat* or mentor* or proctor*):ti,ab	203938
#14	((skill* or abilit* or knowledge or competenc* or proficienc*) near/3 (assess* or monitor* or exam* or credential*)):ti,ab	8481
#15	#12 or #13 or #14	207875
#16	#6 and #11 and #15 in Cochrane Reviews, Cochrane Protocols, Trials	42

KSR Evidence (Internet) (https://ksrevidence.com/ ): to 4 May 2022

Date searched: 4 May 2022

Records found: 6

**Table:**

1	(colorect* or rectal* or rectum* or mesorectal or colon* or sigma* or sigmo* or rectosigm* or bowel* or anal or anus or anorectal or c?ecum or c?ecal or il?eoc?ecal or il?eoc?ecum or duodenum or intestin*) adj4 (surg* or laparoscop* or keyhole or key hole or minimally invasive or MIS or resect* or excis* or dissect*) in All text	937 results
2	(colitis or crohn* or diverticulitis or enterocolitis or diverticulosis or proctalgia or proctocolitis or rectocele or fistula* or fissure* or hernia* or h?emorrhoid*) adj4 (surg* or laparoscop* or keyhole or key hole or "minimal* invasive" or MIS or resect* or excis* or dissect*) in All text	162 results
3	hemicolectom* or colectom* or rectopex* or sigmoidectom* in All text	303 results
4	#1 or #2 or #3 in All text	1221 results
5	robot* or robosurg* in All text	1366 results
6	da vinci or versius in All text	40 results
7	#5 or #6 in All text	1366 results
8	train* or learn* or elearn* or educat* or simulat* or mentor* or proctor* in All text	22531 results
9	(skill* or abilit* or knowledge or competenc* or proficienc*) adj3 (assess* or monitor* or exam* or credential*) in All text	1179 results
10	#8 or #9 in All text	23224 results
11	#4 and #7 and #10 in All text	6 results

An up‐to‐date search was performed on 1 September 2023, and a further 269 results were identified.
